# Impact of COVID-19 pandemic on Saudi medical students’ career choices and perceptions of health specialties: findings from a national cross-sectional study

**DOI:** 10.1186/s12909-022-03224-x

**Published:** 2022-03-14

**Authors:** Firas K. Almarri, Rahaf I. Alshareef, Eman A. Hajr, Fahad Z. Alotabi

**Affiliations:** 1 College of Medicine, Imam Mohammed Ibn Saud Islamic University, Riyadh, Saudi Arabia; 2 Department of Otolaryngology, Imam Mohammad Ibn Saud Islamic University, Riyadh, Saudi Arabia

**Keywords:** COVID-19 effect, Medical education, Medical student, Specialty of choice, Clinical knowledge, Pandemic consequences

## Abstract

**Background:**

The COVID-19 pandemic has had a devastating effect on people across the globe. Its impact on medical students’ education has also been profound. Here, we aimed to comprehensively determine the nature of this impact on their choice of specialty.

**Method:**

A cross-sectional study was conducted among medical students in Saudi Arabia during the pandemic from May to June 2021. Data collected from 1984 medical students were analyzed.

**Results:**

Of the total sample, 810 (40.8%) respondents reported that the pandemic could affect their choice of specialty, with the majority being in the third year (*n* = 235). Across all class-years, the most common reason chosen was the inability to explore specialties of interest (*n* = 539, 66.5%). Another reason cited was the inability to support residency application (*n* = 175, 21.6%). A majority expressed concerns regarding enrollment in research activities. As high as 17.9% (*n* = 356) of the respondents admitted that they were trying to avoid specialty with frontline exposure to COVID-19, while 353 students (17.8%) were considering local training programs only. While examining certainty levels, of the 1174 (59.2%) students who reported not being affected by the pandemic, 924 (78.7%) had a weak certainty level. The majority were in the third (54.8%, *n* = 342) and fourth years (44.8%, *n* = 212).

**Conclusions:**

This study is the first attempt to thoroughly examine the effect of COVID-19 on medical students’ choice of specialty. This effect unfurled in 4 out of 10 surveyed students. Many students reported concerns regarding the inability to explore medical specialties and the inadequacy of obtained clinical knowledge. However, a subsidiary effect was observed among students who were assertive about their choice of specialty. These findings shed new light on the exigency of establishing a career counseling framework designed to meet individual learner needs, thereby galvanizing their morale. Further research could explore the long-term implications of the Saudi Commission for Health Specialties Matching System.

## Background

On March 11, 2020, the World Health Organization declared the coronavirus disease-19 (COVID-19) a public health emergency of global concern [1]. In Saudi Arabia, the Ministry of Health reported the first case of COVID-19 on March 2, 2020. The government response was prompt, culminating in a lockdown that mandated the restriction of medical services, and the closing of schools, colleges, and different educational institutions, including medical universities [2, 3]— to limit the spread of the virus and alleviate the strain on the government’s healthcare system.

The pandemic has inflicted many hurdles on medical students’ education. A key issue is the various limitations of an online virtual format curriculum: students are unable to put their clinical knowledge into action owing to their displacement from clinical rotations and medical school campuses [4]. In these unprecedented times, there are also concerns regarding severe COVID-19 outcomes that extend beyond one’s physical health. Of particular interest is the career perception of medical students and their choice of health specialties, especially newly graduated students and those who are seeking to apply for residency programs during or immediately after the lockdown period. Existing research recognizes the critical role of clinical exposure in galvanizing medical students’ confidence in their future career choices [5–7]. Byrnes et al. [6] who surveyed over 1000 US medical students, found that many students felt that the pandemic would influence their eventual choice of health specialty. Another primary concern, which might have caused an unparalleled experiential gap for senior medical students in Saudi Arabia, is the drastic reduction or prohibition of summer elective rotation opportunities. Recently published editorials and commentaries by medical students highlighted their perturbations regarding elective cancelations owing to COVID-19 [5, 8]. Moreover, clinical exposure through clinical clerkships and elective rotations has a pivotal role in flourishing students’ professional identities [9]. Clinical settings allow students to learn to prioritize patients and strive to be benevolent, and they gain the opportunity to resolve the uncertainty that hovers above their choice of health specialties [7, 9, 10]. Additionally, these disruptions may impose substantial difficulties for students to obtain meaningful letters of recommendation, along with impediments in ameliorating one’s curriculum vitae [11]. Thus, the pandemic continues to be a globally deleterious chapter putting medical students worldwide through trial by fire. Nevertheless, there is a paucity of data describing the effect of COVID-19 on medical students’ academic environment, especially in the Middle East and Gulf countries. Therefore, providing an early snapshot of the possible challenges and concerns faced by Saudi medical students may mitigate their overall experience and bolster the career counseling framework during this unprecedented era. Thus, this study aimed to investigate the impact of COVID-19 on Saudi medical students’ career choices and perceptions of health specialties.

## Methods

### Study design

**Fig. 1:**
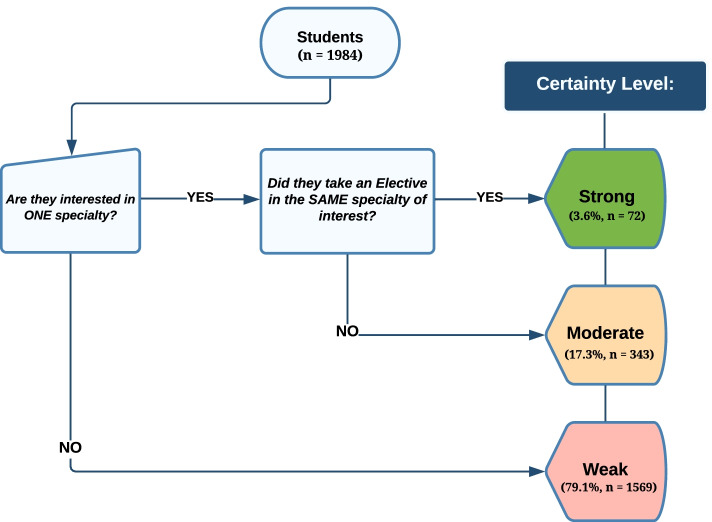
Flowchart demonstrating how certainty levels are distributed based on the specialty of interest and electives

A cross-sectional study was conducted in Saudi Arabia during the pandemic from May to June 2021. A validated English questionnaire was adapted and modified after receiving Dr. Karthik Rajasekaran’s approval, the corresponding author of a study investigating the impact of COVID-19 on medical students’ career perceptions [6]. Since our modified questionnaire additionally inquired about the students’ specialty of interest and completed electives, we created a new variable to confirm the certainty level of their specialty choice, allowing us to gain a detailed understanding of the effects of COVID-19 on medical students (Fig. 1).

The questionnaire was distributed throughout the Kingdom of Saudi Arabia. Prior to this, a pilot study of qualitative methodology was carried out on nine randomly selected medical students to validate the usability and clarity of the questionnaire. These students did not participate in the succeeding survey study. Based on the pilot study analysis, the questionnaire was modified according to our research objectives and academic environment. The final questionnaire was sent out through SurveyMonkey Inc. (San Mateo, California, USA; www.surveymonkey.com), along with a cover letter attached to a consent form. Participation was voluntary, with the option to withdraw at any time. All responses were anonymous, with no tracking of e-mail addresses or any identifying information.

Prior to the study, ethical clearance was obtained from the ethical research committee of the Institutional Review Board (IRB) of Imam Mohammed Ibn Saud Islamic University (IMSIU), Riyadh, Saudi Arabia, wherein they reviewed and approved this project (HAPO-01-R-001, Project No. 72–2021).

### Survey distribution

The survey was distributed nationally after generating a list containing 38 medical colleges (government and private colleges) using a multifaceted approach. A recruitment form for data collectors (medical students) across all regions of Saudi Arabia (Central, West, East, South, and North) was sent out through official e-mail addresses of each university or by contacting the students’ club of each university. The recruitment form received over 400 responses. Thirty respondents were carefully chosen to participate in our study as data collectors. We allocated 5–8 students from each region, with equal gender representation and different levels, to facilitate normal distribution across all variables. A well-structured handbook with a description of and clear instructions for the study was delivered to all data collectors, along with the availability of 24/7 technical team support. The data collectors then distributed the questionnaire among their peers through emails, social media platforms, or on-site distribution. The research team actively monitored the data collectors to produce a more representative sample from each region.

### Statistical analysis

IBM SPSS version 19 was used to perform statistical analyses. Descriptive statistics were used to outline the characteristics of responders using frequencies, and percentages for categorical variables. Chi-squared test was used to determine the association of variables based on certainty levels among clinical years. *P* values less than .050 were considered to be statistically significant.

## Results

### Demographic characteristics of respondents

**Fig. 2:**
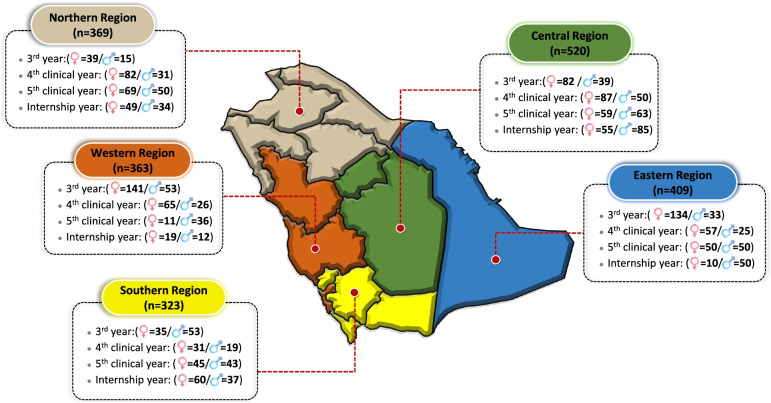
Demographic characteristics of respondents

A total of 1984 out of 2245 medical students responded appropriately to all sections of the structured questionnaire, with a response rate of 88%. The students were from different regions of Saudi Arabia: the highest percentages were from the Central (26.2%) and the Eastern regions (20.6%), followed by the Northern (18.6%) and the Western (18.3%) regions and finally the southern regions (16.3%). Women respondents were slightly predominant in the sample (59.5%) than men (40.5%). A greater number of the respondents were in the third year (31.5%) and the fifth clinical year (24%), followed by the fourth clinical year (23.8%) and the internship year (20.7%) (Fig. 2).

### Clinical characteristics of respondents

In terms of the specialty certainty levels, only 72 (3.6%) students showed a strong certainty level, while 343 (17.3%) reported a moderate certainty level, and a majority of 1569 students (79.1%) had a weak certainty level. A total of 798 (40%) of the respondents (*n* = 798) had not started any core clinical rotation, a majority of them were in the third year 504 (63.2%); only 471 (23.7%) had completed all the core clinical rotations, with the highest percentage in the internship year 215 (45.6%). The remaining 715 (36%) respondents reported having completed a few clinical rotations; most of them were in the fourth clinical year 273 (38.2%). Internal medicine and surgery were the most completed specialties in clinical rotations by students (*n* = 526, 26.5% and *n* = 503, 25.3%, respectively), followed by pediatrics 332 (16.7%) and obstetrics/gynecology 318 (16%). The least completed specialties were dermatology 147 (7.4%) and orthopedic surgery 154 (7.8%).

Regarding the specialty/specialties of interest, internal medicine was the most preferred 634 (32%), followed by general surgery 552 (27%), and then emergency medicine and family medicine with almost equivalent results of 512 (25.8%) and 508 (25.6%), respectively. The least preferred specialties were pathology 112 (5.6%), pediatric neurology 98 (4.9%), and physical medicine and rehabilitation 76 (3.8%).

A total of 825 (41.6%) respondents did not undergo any elective rotation. Meanwhile, 464 (23.4%) reported taking elective rotations in their specialty or specialties of interest. Of those 464 respondents, half of them were students in the internship year 230 (49.6%). Interestingly, 669 (33.7%) of respondents expressed their desire to undergo elective rotations but were unable to do so due to the pandemic. Majority of them 203 (31.5%) were third year students. Among the 26 students who reported other reasons, 17 reported that they would take an elective this summer.

**Fig. 3:**
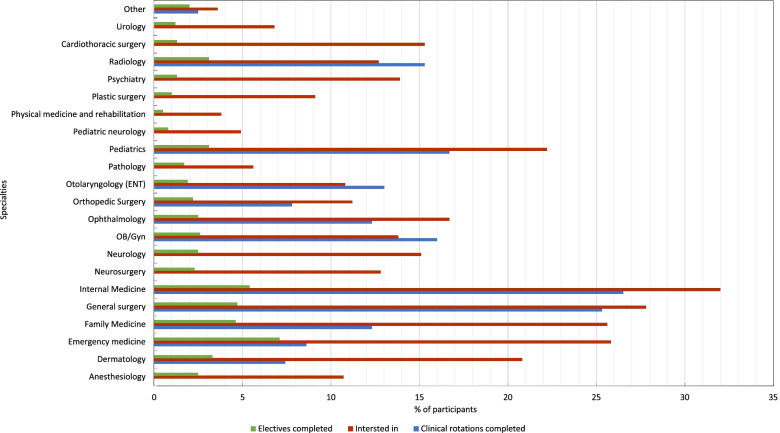
Different specialties completed in clinical rotations, specialties of interest, and specialties completed in electives

In contrast to completed clinical rotations and specialties of interest, emergency medicine was the most frequently reported in completed electives 141 (7.1%), followed by internal medicine 108 (5.4%), then general surgery and family medicine (*n* = 93, 4.7% and *n* = 91, 4.6%, respectively). Figure 3 summarizes the percentages of respondents regarding different specialties completed in clinical rotations, specialties of interest, and specialties completed in electives.

### COVID-19 effect

#### COVID-19 effect on specialty choice

More than half of the respondents 1174 (59.2%) reported that the pandemic did not affect their medical specialty choice: a majority of these respondents were third year students 389 (33.1%). Significant differences were noted in the certainty levels of students in the third year (*P* = .007) and internship year (*P* = .032). Of the 72 students with strong certainty levels, 20 (27.8%) believed that the pandemic affected their specialty choice; however, almost twice this proportion reported similar results among those with moderate 145/343 (42.3%) and weak 645/1569 (41.1%) certainty levels.

By subgrouping according to both certainty level and medical year, the highest percentage of this disagreement within a strong certainty level was noted in students in the internship year 40 (9.7%). With regard to third year students, more students with strong certainty levels agreed on the effect of the pandemic on their medical specialty choice (*n* = 4, 0.6% versus *n* = 1, 0.2%), while a greater number of these students with weak certainty levels disagreed (*n* = 342, 54.8% versus *n* = 187, 30%); almost the same percentages existed among students with moderate certainty levels. However, in Table 1, more students in the fourth clinical year within all three certainty levels disagreed. Similarly, in the fifth clinical year and the internship year, more students within all three certainty levels disagreed, as depicted in Table 1. Among 810 students who thought COVID-19 pandemic could affect their choice, 539 (66.5%) students felt that they might not have the opportunity to explore their specialties of interest. Another 280 (34.6%) respondents reported that they had discovered new interests or priorities; interestingly, a large proportion of them were third year students 87 (31.1%) with the lowest proportion in the internship year 49 (17.5%). About 175 (21.6%) thought that they no longer had the ability to support their application. Similar percentages were noted among all certainty level groups. A total of 38 (1.9%) respondents reported that they were concerned about research in their applications. A similar number of respondents were concerned about outside rotations and taking board examinations (*n* = 35, 1.8% and *n* = 34, 1.7%, respectively) (Table 1).

**Table 1:** Questionnaire response, illustrating the effect of COVID-19 subgrouped by certainty choice level and medical year

Item	Total (***n*** = 1984)			Chi square	*P*	3rd year (***n*** = 624)			Chi square	*P*	4th year (***n*** = 473)		
	Strong Level	Moderate level	Weak level			Strong Level	Moderate level	Weak level			Strong Level	Moderate level	Weak level
	N (%)^**a**^	N (%)^**a**^	N (%)^**a**^			N (%)	N (%)	N (%)			N (%)	N (%)	N (%)
	72 (3.6%)	343 (17.3%)	1569 (79.1%)										
***Do you think the COVID-19 pandemic will affect your choice of specialty?***													
Yes	20 (2.5%)	145 (17.9%)	645 (79.6%)	5.4	.066	4 (0.6%)	44 (7.1%)	187 (30%)	9.9	**.007**	4 (0.8%)	34 (7.2%)	169 (35.7%)
No	52 (4.4%)	198 (16.9%)	924 (78.7%)			1 (0.2%)	46 (7.4%)	342 (54.8%)			6 (1.3%)	48 (10.1%)	212 (44.8%)
***If you answered Yes to the previous question, please tell us why.***^***b***^													
I may not have the opportunity to explore my specialty or specialties of interest	13 (2.4%)	82 (15.2%)	444 (82.4%)	5.9	.053	3 (0.48%)	22 (3.5%)	117 (18.75%)	4.2	.122	1 (0.2%)	18 (3.8%)	113 (24%)
I have discovered new interests or priorities	5 (1.8%)	48 (17.1%)	227 (81.1%)	3.2	.200	1 (0.2%)	15 (2.4%)	71 (11.4%)	0.8	.661	3 (0.6%)	12 (2.5%)	64 (13.5%)
I no longer have the ability to support my application	3 (1.7%)	27 (15.4%)	145 (82.9%)	2.7	.263	0	8 (1.3%)	46 (7.4%)	0.5	.786	0	7 (1.5%)	37 (7.8%)
Other	1 (7.7%)	1 (7.7%)	11 (84.6%)			0	0	5 (0.8%)			0	0	4 (0.8%)
***What part(s) of your application are you concerned about?***^***b***^													
Away rotations	0	6 (17.1%)	29 (82.9%)	1.4	.507	0	0	10 (1.6%)	1.8	.402	0	1 (0.2%)	10 (2.1%)
Research	1 (2.6%)	10 (26.3%)	27 (71.1%)	2.2	.325	0	4 (0.6%)	9 (1.4%)	2.9	.229	0	2 (0.4%)	8 (1.7%)
Letter of Recommendation	1 (5%)	4 (20%)	15 (75%)	0.2	.890	0	0	7 (1.1%)	1.3	.530	0	1 (0.2%)	2 (0.4%)
Networking	1(4.5%)	4(18.2%)	17(77.3%)	0.1	.965	0	3 (0.48%)	11 (1.76%)	0.7	.716	0	0	4 (0.8%)
Taking board examination	1 (2.9)	6(17.6%)	27(79.4%)	0.0	.976	0	0	11 (1.76%)	2.0	.366	0	4 (0.8%)	6 (1.3%)
Other	1 (33.3%)	1 (33.3%)	1 (33.3%)			0	0	1 (0.2%)			0	0	0
***Do you think that the COVID-19 pandemic changed your perception toward future medical specialty and career?***													
Yes	25 (2.9%)	162 (18.5%)	687 (78.6%)	4.0	.136	4 (0.6%)	48 (7.7%)	246 (39.4%)	3.5	.170	5 (1.1%)	44 (9.3%)	180 (38%)
No	47 (4.2%)	181 (16.3%)	882 (79.5%)			1 (0.2%)	42 (6.7%)	283 (45.35%)			5 (1.1%)	38 (8%)	201 (42.5%)
***What aspect of your perception changed?***^***b***^													
Considering local training programs only (avoid abroad applications for medical training)	15 (4.2%)	55 (15.6%)	283 (80.2%)	1.2	.537	3 (0.48%)	17 (2.7%)	95 (15.2%)	5.8	.054	3 (0.6%)	11 (2.3%)	73 (15.4%)
Trying to avoid the specialty with frontline exposure to the pandemic (e.g., ER, anesthesia)	4 (1.1%)	66 (18.5%)	286 (80.3%)	8.0	**.018**	1 (0.2%)	24 (3.8%)	96 (15.38%)	3.6	.168	0	15 (3.2%)	82 (17.3%)
Considering specialty of basic science without clinical exposure	2 (1.1%)	30 (16.9%)	145 (81.9%)	3.6	.169	0	14 (2.2%)	61 (9.8%)	1.9	.393	1 (0.2%)	8 (1.7%)	41 (8.7%)
Considering specialty with short residency training	2 (1.1%)	34 (18.4%)	149 (80.5%)	3.9	.146	0	12 (1.9%)	58 (9.3%)	1.1	.586	1 (0.2%)	10 (2.1%)	40 (8.5%)
Working outside the field (away from medical specialties e.g., business field)	2 (1.3%)	24 (15.1%)	133 (83.6%)	3.6	.164	0	6 (0.96%)	54 (8.7%)	1.6	.439	0	5 (1.1%)	30 (6.3%)
Considering specialties with frontline exposure	5 (2.5%)	26 (13.1%)	168 (84.4%)	3.8	.147	0	7 (1.1%)	76 (12.2%)	3.7	.160	1 (0.2%)	8 (1.7%)	37 (7.8%)
Other	1 (3.2%)	6 (19.4%)	24 (77.4%)			0	1 (0.2%)	13 (2.1%)			0	3 (0.6%)	3 (0.6%)
***Has the COVID-19 pandemic made you more likely to take an extra year in clinical service before applying to residency?***													
No	56 (3.9%)	231 (16.1%)	1149 (80.0%)	6.0	.051	4 (0.6%)	33 (5.3%)	124 (19.9%)	14.8	**.001**	2 (0.4%)	27 (5.7%)	113 (24%)
Yes	16 (2.9%)	112 (20.4%)	420 (76.6%)			1 (0.2%)	57 (9.1%)	405 (64.9%)			8 (1.7%)	55 (11.6%)	268 (56.7%)
***If you answered Yes to the previous question, please tell us why.***													
It would give me more time to explore different specialties	10 (3.2%)	55 (17.6%)	247 (79.2%)	0.2	.901	3 (0.48%)	14 (2.2%)	78 (12.5%)	7.9	**.020**	1 (0.2%)	13 (2.7%)	68 (14.4%)
It would make me more likely to match to my satisfaction	3 (1.3%)	39 (16.5%)	195 (82.3%)	4.6	.100	1 (0.2%)	17 (2.7%)	55 (0.8%)	5.7	.058	0	7 (1.5%)	48 (10.1%)
I may not be able to meet the requirements for residency acceptance	3 (1.4%)	39 (17.7%)	178 (80.9%)	3.6	.163	0	13 (2.1%)	57 (9.1%)	1.7	.432	0	8 (1.7%)	39 (8.2%)
I want to use the extra year to explore new interests that developed during the pandemic	3 (1.8%)	23 (13.7%)	142 (84.5%)	3.7	.151	0	7 (1.1%)	61 (9.8%)	1.7	.421	1 (0.2%)	6 (1.3%)	32 (6.7%)
Other	0	1 (11.1%)	8 (88.9%)			0	0	4 (0.6%)			0	0	1 (0.2%)

Item	Chi square	*P*	5th year (***n*** = 476)			Chi square	*P*	Internship year (***n*** = 411)			Chi square	*P*
			Strong Level	Moderate level	Weak level			Strong Level	Moderate level	Weak level		
			N (%)	N (%)	N (%)			N (%)	N (%)	N (%)		
***Do you think the COVID-19 pandemic will affect your choice of specialty?***												
Yes	0.3	.866	2 (0.4%)	33 (7%)	182 (38.2%)	2.1	.353	10 (2.4%)	34 (8.3%)	107 (26%)	6.9	**.032**
No			5 (1%)	49 (10.3%)	205 (43%)			40 (9.7%)	55 (13.4%)	165 (40.1%)		
***If you answered Yes to the previous question, please tell us why.***^***b***^												
I may not have the opportunity to explore my specialty or specialties of interest	3.6	.164	2 (0.4%)	21 (4.4%)	136 (28.6%)	2.8	.242	7 (1.7%)	21 (5.1%)	78 (19%)	5.0	.080
I have discovered new interests or priorities	1.5	.466	0	11 (2.3%)	54 (11.3%)	1.1	.565	1 (0.2%)	10 (2.4%)	38 (9.2%)	5.8	.055
I no longer have the ability to support my application	1.2	.560	0	7 (1.5%)	37 (7.8%)	0.8	.668	3 (0.7%)	5 (1.2%)	25 (6%)	1.5	.478
Other			0	0	1 (0.2%)			1 (0.2%)	1 (0.2%)	1 (0.2%)		
***What part(s) of your application are you concerned about?***^***b***^												
Away rotations	0.8	.660	0	3 (0.6%)	6 (1.26%)	1.8	.415	0	2 (0.5%)	3 (0.7%)	1.4	.489
Research	0.3	.897	0	3 (0.6%)	7 (1.5%)	1.3	.528	1 (0.2%)	1 (0.2%)	3 (0.7%)	0.3	.865
Letter of Recommendation	0.6	.748	0	2 (0.4%)	3 (0.6%)	1.9	.391	1 (0.2%)	1 (0.2%)	3 (0.7%)	0.3	.865
Networking	1.0	.614	0	0	1 (0.2%)	0.2	.891	1 (0.2%)	1 (0.2%)	1 (0.2%)	1.8	.407
Taking board examination	3.8	.151	0	1 (0.2%)	6 (1.26%)	0.2	.924	1 (0.2%)	1 (0.2%)	4 (1%)	0.2	.918
Other			0	0	0			1 (0.2%)	1 (0.2%)	0		
***Do you think that the COVID-19 pandemic changed your perception toward future medical specialty and career?***												
Yes	1.1	.571	1 (0.2%)	31 (6.5%)	153 (32.1%)	1.9	.388	15 (3.6%)	39 (9.5%)	108 (26.3%)	2.6	.274
No			6 (1.26%)	51 (10.7%)	234 (49%)			35 (8.5%)	50 (12.2%)	164 (40%)		
***What aspect of your perception changed?***^***b***^												
Considering local training programs only (avoid abroad applications for medical training)	2.4	.301	1 (0.2%)	12 (2.5%)	64 (13.4%)	0.2	.905	8 (1.9%)	15 (3.6%)	51 (12.4%)	0.3	.853
Trying to avoid the specialty with frontline exposure to the pandemic (e.g., ER, anesthesia)	3.1	.216	0	15 (3.2%)	64 (13.4%)	1.6	.457	3 (0.7%)	12 (2.9%)	44 (10.7%)	3.6	.163
Considering specialty of basic science without clinical exposure	0.1	.963	0	3 (0.6%)	32 (6.7%)	2.7	.263	1 (0.2%)	5 (1.2%)	11 (2.7%)	1.1	.584
Considering specialty with short residency training	0.2	.901	0	4 (0.8%)	29 (6.1%)	1.2	.536	1 (0.2%)	8 (1.9%)	22 (5.4%)	2.6	.274
Working outside the field (away from medical specialties e.g., business field)	1.1	.569	0	7 (1.5%)	26 (5.5%)	0.9	.645	2 (0.5%)	6 (1.5%)	23 (6%)	1.3	.520
Considering specialties with frontline exposure	0.0	.991	0	4 (0.8%)	35 (7.5%)	2.2	.334	4 (1%)	7 (1.7%)	20 (4.9%)	0.0	.979
Other			0	2 (0.4%)	4 (0.8%)			1 (0.2%)	0	4 (1%)		
***Has the COVID-19 pandemic made you more likely to take an extra year in clinical service before applying to residency?***												
No	0.8	.660	1 (0.2%)	27 (5.7%)	112 (23.5%)	1.3	.522	9 (2.2%)	25 (6.1%)	71 (17.3%)	1.8	.398
Yes			6 (1.26%)	55 (11.6%)	275 (57.8%)			41 (10%)	46 (11.2%)	201 (49%)		
***If you answered Yes to the previous question, please tell us why.***												
It would give me more time to explore different specialties	0.6	.751	1 (0.2%)	15 (3.2%)	65 (13.7%)	0.1	.930	5 (1.2%)	13 (3.2%)	36 (8.8%)	0.6	.740
It would make me more likely to match to my satisfaction	2.4	.297	0	8 (1.7%)	52 (11%)	1.9	.395	2 (0.5%)	7 (1.7%)	40 (9.7%)	6.4	**.041**
I may not be able to meet the requirements for residency acceptance	1.1	.564	0	6 (1.26%)	48 (10%)	2.6	.266	3 (0.7%)	12 (2.9%)	34 (8.3%)	0.2	.375
I want to use the extra year to explore new interests that developed during the pandemic	0.1	.990	0	4 (0.8%)	30 (6.3%)	1.4	.499	2 (0.5%)	6 (1.5%)	19 (4.6%)	0.6	.734
Other			0	1 (0.2%)	2 (0.4%)			0	0	1 (0.2%)		

^a^Percentages were calculated based on the total of number of students who responded to the items

^b^Respondents could select more than one answer

#### COVID-19 effect on future medical specialty and career perceptions

Regarding the choice of specialty, about 1110 (55.9%) of respondents disagreed that the pandemic changed their perceptions toward future medical specialty and career. Among students with a strong certainty level, disagreement was high in students in the fifth and the internship years (*n* = 6, 1.26% versus *n* = 1, 0.2% and *n* = 35, 8.5% versus *n* = 15, 3.6%, respectively). While the percentages of students in agreement and disagreement were equal among students in the fourth year (*n* = 5, 1.1%), the percentage of students in agreement was higher in the third year (*n* = 4, 0.6% versus *n* = 1, 0.2%). More students agreed on the effect of COVID-19 with a moderate certainty level in both the third and the fourth years (*n* = 48, 7.7% versus *n* = 42, 6.7% and *n* = 44, 9.3% versus *n* = 38, 8%, respectively).

Consequently, only 356 respondents (17.9%) admitted that they were trying to avoid the specialties with frontline exposure to the pandemic, for example, ER and anesthesia, with significantly higher participants in weak certainty level 286 (80.3%), and 353 respondents (17.8%) were considering only local training programs in an attempt to avoid abroad applications for medical training. In addition, 185 (9.3%) of the respondents reported considering a specialty with short residency training. Some students even considered the specialty of basic science without clinical exposure, and working outside the medical field (*n* = 177, 8.9% and *n* = 159, 8%, respectively), for example, the business field. Most of these students were from the third year (*n* = 75, 42.4% and *n* = 60, 37.7%, respectively). Meanwhile, 199 (10%) respondents were considering specialties with frontline exposure, out of which the highest number was from the third year 83 (41.7%). Contrary to the highest number of students in both the moderate and weak certainty level groups, who attempted to avoid specialties with frontline exposure (*n* = 66/343, 19% and *n* = 286/1569, 18%), the highest number of students with strong certainty level chose to consider local training programs only 15/72 (20.8%).

More than half of the respondents 1436 (72.4%) disagreed that the pandemic made them more likely to take up an extra year in clinical service before applying for residency. Significant differences were observed among third year students’ certainty levels (*P* = .001). However, the 548 (27.6) respondents who agreed to this explained that this extra year would give them more time to explore different specialties 312 (57%) and to match to their satisfaction 237 (43.2%). A total of 220 (40.1%) respondents reported that they might not meet the requirements for residency acceptance and 168 (30.7%) respondents wanted to use the extra year to explore new interests that they developed during the pandemic. Among the nine students who reported other reasons for taking an additional year, five reported trepidation and timidity with regard to their quality of clinical knowledge, which was disadvantaged by the lack of clinical exposure due to COVID-19 (Table 1).

### Activities while away from the hospital and clinical rotations

**Fig. 4:**
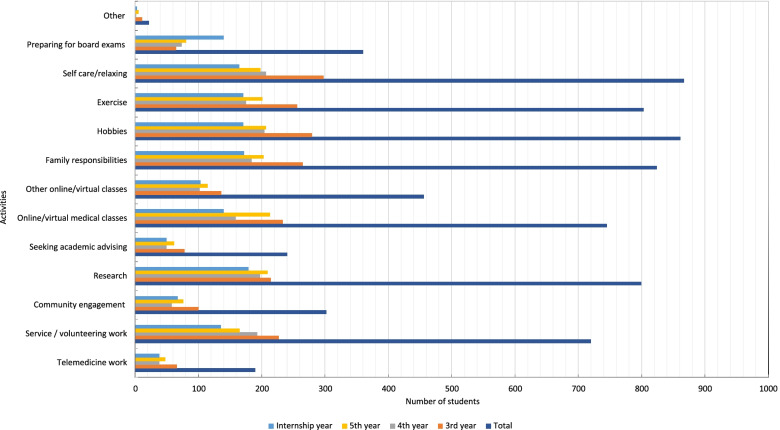
Activities students engaged in during the pandemic away from the medical school and hospital

In terms of activities away from the hospital during the pandemic, self-care/relaxing was reported by 867 (43.7%) respondents, followed by hobbies 861 (43.4%). Subsequently, family responsibilities, exercise, and research were reported with almost similar percentages (*n* = 824, 41.5%, *n* = 803, 40.5% and *n* = 799, 40.3%, respectively). Online/virtual classes through medical school were reported by 745 (37.6%); other online/virtual classes were reported by 456 (23%). Service/volunteering work and community engagement or organizing were reported by 720 (36.3%) and 302 (15.2%), respectively. Other medical-related activities were noted, such as telemedicine work 190 (9.6%), seeking academic advising 240 (12.1%), and preparing for board exams 360 (18.1%) (Fig. 4).

## Discussion

Choosing a medical specialty remains one of the biggest challenges for most medical students as it requires extensive knowledge and research into available opportunities and residency programs. In this study, we explored the impact of COVID-19 on medical students’ career choices and perceptions of health specialties in Saudi Arabia. Amid the unprecedented challenges posed by the pandemic, two-fifths of our respondents conveyed that their specialty choice would be affected by it. However, in their study conducted across the US during April 13–30, 2020, Byrnes et al. [5] found that only one-fifth of the respondents expressed that their specialty choice would be affected by COVID-19. This could be attributed to the study time difference, as Saudi students were exposed to a lengthier pandemic restriction time compared to US students. As expected, among the 810 (40.8%) medical students in our sample whose specialty choices were affected, a majority indicated concerns related to not having the opportunity or the time to explore their specialties of interest. These results could have been obtained owing to the drastic reduction of summer elective rotation opportunities offered to medical students, in line with the precautionary measures imposed by the Ministry of Health in Saudi Arabia. Almost 669 (33.7%) of our respondents reported their desire to take a summer elective but not managing to do so. Exposure to a specialty in a clinical environment is integral in developing a physician’s professional identity. Previous studies show that such exposure will certainly boost medical students’ confidence regarding their possible future career choice [7, 12, 13]. Interestingly, 280 (34.6%) of the respondents, whose specialty choices were affected by the pandemic, reported discovering new interests or priorities. A majority of them (*n* = 87, 31.1%) were third year students, while only 49 (17.5%) were internship year students. A possible explanation for this could be that students in the internship year are limited by the time required to develop new interests, as they would have already decided upon their specialty of choice (considering how far they have completed their course), as opposed to third year students who may have not made their decision yet.

In Fig. 3, it can be seen that the percentage of students interested in a specialty exceeded the percentage of students who completed core clinical rotations or took elective rotations for almost all the specialties. This discrepancy could be attributed to the high number of respondents from the third year, who may not have had the chance to complete all the clinical rotations or take an elective. This implies that it may not be possible to clearly examine the effect of COVID-19 on students’ specialty choices. Therefore, a divergence in the sincerity of the impact of COVID-19 on students might arise. Hence, the results of the previous study by Byrnes et al. [5] should be interpreted with caution.

It is also imperative to examine the students’ seriousness in choosing medical specialties. To do so, one can filter a student’s specialty of interest and link it to whether or not the student completed an elective in the same specialty. Subsequently, we can subgroup the respondents by different levels of certainty about their specialty of choice (Fig. 1). The 924 students, who reported that they were not affected by the pandemic, were, in fact, at a weak certainty level. Majority of students were in the third and fourth clinical years—342 (54.8%) and 212 (44.8%), respectively. Thus, the reason they mentioned that they were not affected by the pandemic might be strongly linked to their uncertainty regarding the specialty choice in the first place. Compared to the 52 (4.4%) students within the strong certainty level who disagreed on the effect of COVID-19 on their specialty of choice, 40 (9.7%) of them were, in fact, in the internship year. This is clearly because of the fact they were close to applying for residency programs, and therefore, the pandemic did not affect their seriousness regarding their specialty of choice.

With regard to the COVID-19 effect on future medical specialty and career perceptions, 874 (41.1%) of our respondents agreed to being affected by the pandemic. When asked what aspect of their perception changed, most of them reported that they were considering a specialty without frontline exposure to COVID-19 and direct contact with a patient. The reason for this is not apparent, but it might be related with the higher infection and mortality rates caused by COVID-19 compared to previous pandemics faced by healthcare personnel. A lower percentage was recorded for the choice of turnover to other non-medical fields (*n* = 159, 8.0%), yet, this number is impactful to the medical field. This finding broadly supports the work of Carla Zi Cai, who reported high turnover rates of medical students due to the fear of COVID-19 in China [13].

A fair number of students who decided to take an extra year in clinical service before applying for residency, reported independently through comment boxes, their fears regarding serious impediments in their level of clinical knowledge. Many of them reported not feeling ready or competent to treat and handle patients. This necessitates the creation of a structural framework to target, address, and meet medical students’ needs early on.

Our results showed that students were engaging in different activities, the most important being research. About 799 (40.3%) of the students in our sample were engaged in ongoing research projects to enrich their research experience during the pandemic. Other activities such as community and volunteering work, personal hobbies, and spending more time with family have been reported by previous studies [5, 7, 14]. A fair percentage of students utilized the time to attend extracurricular virtual classes and courses which may help them fill some gaps that resulted from the scarcity of clinical exposure.

This study has several strengths, including a large sample size, diverse geographic distribution, and a coherent and representative sample. However, there are a few limitations to consider; most notably, the risk of response bias. Those willing to respond to the questionnaires might have different opinions and perspectives toward the pandemic than those who were not. Another inevitable limitation that came from ensuring maximum anonymity was that our questionnaire did not ask respondents to mention their medical school’s name (only the region where they attended was asked). Another uncontrolled factor is assessing the reliability and validity of medical students’ certainty levels regarding their choice of specialty*.* Although our results predominantly substantiate a conceptual framework of the effect of COVID-19 on specialty choice, these findings should be generalized with caution. Despite these exciting results, questions remain about the long-term implications of the COVID-19 pandemic on the Saudi Commission for Health Specialties Matching System, and whether the immunity gained by the public through the vaccine campaigns might inflict a different perception and pathway toward future career choices for medical students.

## Conclusion

COVID-19 has had a severe impact on medical students’ clinical education across Saudi Arabia. Our study demonstrated a clear effect of the pandemic on medical students’ career choices and perceptions. Four out of ten respondents agreed that their specialty choices were affected by the pandemic The inability to explore medical specialties and the lack of proper clinical education and exposure are the most pressing concerns among medical students. The present study provides the first comprehensive assessment of the effect of COVID-19 on students’ career choices. Therefore, we can conclude that the more certain students are about their specialty of choice, the less negative is the impact of the pandemic on them.

## Data Availability

The datasets used and/ or analyzed from the current study are available from the corresponding author on reasonable request.
